# Antiinflammatory Actions of Klotho: Implications for Therapy of Diabetic Nephropathy

**DOI:** 10.3390/ijms22020956

**Published:** 2021-01-19

**Authors:** Marlena Typiak, Agnieszka Piwkowska

**Affiliations:** 1 Laboratory of Molecular and Cellular Nephrology, Mossakowski Medical Research Centre, Polish Academy of Sciences, Wita Stwosza 63, 80-308 Gdansk, Poland; apiwkowska@imdik.pan.pl; 2 Department of Molecular Biotechnology, Faculty of Chemistry, University of Gdansk, Wita Stwosza 63, 80-308 Gdansk, Poland

**Keywords:** Klotho protein, diabetes mellitus, diabetic nephropathy, inflammation, diagnosis, therapy

## Abstract

Klotho was initially introduced as an antiaging molecule. Klotho deficiency significantly reduces lifespan, and its overexpression extends it and protects against various pathological phenotypes, especially renal disease. It was shown to regulate phosphate and calcium metabolism, protect against oxidative stress, downregulate apoptosis, and have antiinflammatory and antifibrotic properties. The course of diabetes mellitus and diabetic nephropathy resembles premature cellular senescence and causes the activation of various proinflammatory and profibrotic processes. Klotho was shown to exert many beneficial effects in these disorders. The expression of Klotho protein is downregulated in early stages of inflammation and diabetic nephropathy by proinflammatory factors. Therefore, its therapeutic effects are diminished in this disorder. Significantly lower urine levels of Klotho may serve as an early biomarker of renal involvement in diabetes mellitus. Recombinant Klotho administration and Klotho overexpression may have immunotherapeutic potential for the treatment of both diabetes and diabetic nephropathy. Therefore, the current manuscript aims to characterize immunopathologies occurring in diabetes and diabetic nephropathy, and tries to match them with antiinflammatory actions of Klotho. It also gives reasons for Klotho to be used in diagnostics and immunotherapy of these disorders.

## 1. Diabetes Mellitus and Diabetic Nephropathy

Diabetes mellitus (DM) is a serious, chronic disease that is characterized by high blood glucose levels, resulting in aberrant insulin production and lower sensitivity of acceptor cells of this hormone. The prevalence of DM is increasing worldwide, and its complications are one of the leading causes of mortality from noncommunicable diseases [1,2]. Due to the high prevalence of diabetes and because 30–40% of diabetic patients (both type 1 (T1DM) and type 2 (T2DM) DM) develop kidney dysfunction, diabetic nephropathy is the main cause of end-stage renal disease worldwide.

Proper kidney function is essential for maintaining body homeostasis through selective plasma filtration. Blood filtration occurs in the cortex of the kidney, in the glomerulus of the nephron, which apart from a glomerulus also consists of the proximal tubule, Henle’s loop, and distal tubule. Blood flows to glomeruli and is filtered by a three-layer structure (i.e., filtration barrier): pores between endothelial cells of blood capillaries, the glomerular basement membrane (created by endothelial cells and podocytes), and spaces between neighboring podocytes (i.e., slit diaphragms). A podocyte cell consists of a cell body, major processes, and foot processes that branch out from major processes and is tightly laid on blood capillaries to form slit diaphragms. The structure of the glomerulus is also supported by mesangial cells.

During the course of diabetes and diabetic kidney disease, many metabolic and hemodynamic stresses occur, among others hypoxia and hyperfiltration [3,4]. The disruption of body homeostasis in diabetic patients is caused by a hyperglycemia-induced increase in the generation of reactive oxygen species (ROS). This leads to the oxidation of DNA, proteins, and lipids and subsequent organ damage and the apoptosis of podocytes, which are non-proliferating cells [5]. Another change in protein patterns in diabetes patients includes protein hyperglycation through excessive blood glucose levels. The exacerbation of this process causes the production of irreversible advanced glycation end products (AGEs), which are long lasting, accumulate to high levels in tissues during age-related chronic diseases, stimulate the immune system, and are proposed to serve as an early predictor of kidney complications in diabetes [6]. Furthermore, the aforementioned metabolic and hemodynamic stresses during diabetes and diabetic nephropathy can cause cellular dysfunction and damage and stimulate an inflammatory response and subsequent fibrosis, resulting in renal injury. Pathological processes of diabetic nephropathy resemble kidney injury through cellular senescence [7], with a shortening of telomeres, DNA damage, epigenetic alterations, changes in protein patterns, mitophagy deficiencies, the downregulation of Klotho expression, inflammation, the activation of profibrotic Wnt/β-catenin signaling, and the accumulation of uremic toxins (e.g., indoxyl sulfate) that further reduce renal Klotho expression and intensify fibrosis [8,9,10,11,12].

## 2. Klotho Protein and Its Tissue Expression

α-Klotho was discovered in 1997 and simply named Klotho because no other Klotho proteins were identified at that time. It was shown to be an antiaging molecule. Mice that exhibited Klotho deficiency presented a premature aging phenotype, whereas Klotho overexpression extended their lifespan by up to 30% and protected them against many pathological phenotypes, especially renal disease [13,14].

Klotho is expressed mostly in the kidneys and choroid plexus in the brain. Its expression was also detected in the pituitary, parathyroid glands, the heart, and reproductive organs, with low levels in other tissues [13]. In the kidneys, Klotho is present in podocytes, the (mostly) apical and basolateral membrane, and intracellularly in cells that comprise the proximal tubule of the nephron. Klotho is also shed to the proximal tubule lumen [15,16]. Kidney cells are the main source of soluble Klotho, but it can also be shed from ependymal cells of the choroid plexus [17]. Soluble Klotho was detected in blood, urine, and cerebrospinal fluid and is considered to be a hormone that exerts beneficial systemic effects [13]. The reason why soluble Klotho can target so many tissues and affect various signaling pathways is currently unknown because no specific receptor for soluble Klotho has yet been identified. The pleiotropic effects of soluble Klotho are hypothesized to be based on its capacity to bind sialic acid and target monogangliosides (e.g., monosialotetrahexosylganglioside—GM1 and monosialodihexosylganglioside—GM3) that are enriched in lipid rafts of cell membranes. Soluble Klotho can also interact with numerous intracellular proteins [17].

## 3. Structure and Function of Klotho

Two or three forms of α-Klotho protein exist. Membrane-bound Klotho is a 130 kDa single-pass transmembrane protein that is encoded by a *KL* gene. The extracellular domain of the Klotho protein can be cleaved and separated from the cell membrane by the metalloproteinases ADAM-10 and ADAM-17 (ADAM—a disintegrin and metalloproteinase domain-containing protein) and β-secretase 1 (BACE1). This proteolytic cleavage by ADAM-10 and ADAM-17 can be stimulated inter alia by insulin. The extracellular domain of Klotho contains two homologous repeat sequences (KL1 and KL2), which are separated from each other by unknown proteases. Both the whole released extracellular domain and separated KL1 and KL2 are referred to as soluble Klotho particles [13]. Another, alternative transcript of the *KL* gene that possesses a STOP codon was thought to result in the production of a shorter, secretory form of the protein [18]. However, according to some researchers, this transcript is degraded and not translated into a protein product. Moreover, splicing of the two (standard functional and alternative) mRNA transcripts of Klotho in humans is dysregulated especially in acute kidney injury (AKI), favoring the nonfunctional alternative transcript. This might contribute to the decrease in the expression of functional Klotho protein also in diabetic nephropathy [14].

Both transmembrane Klotho and soluble Klotho serve as coreceptor proteins for fibroblast growth factor (FGF) 23 (binding to FGF receptors 1–4), which promotes phosphaturia to control phosphate metabolism [14]. Klotho also functions independently to regulate many signaling pathways because it is evolutionarily older than FGF23 [19]. Klotho regulates not only phosphate metabolism but also calcium metabolism. Furthermore, it inhibits the insulin/insulin-like growth factor 1 (IGF-1) signaling pathway and activates forkhead box (FoxO) transcription factors, resulting in the production of antioxidant enzymes (e.g., catalase and manganese-dependent superoxide dismutase [20]) and reduction of oxidative stress through the removal of ROS, thereby downregulating apoptosis. It also suppresses tumor necrosis factor α (TNF-α)-induced oxidative damage and prevents the translocation of nuclear factor κ-light-chain-enhancer of activated B cells (NF-κB), a transcription factor of many proinflammatory genes, including TNF-α, to the nucleus. Klotho also suppresses the profibrotic transforming growth factor β1 (TGF-β1) and Wnt/β-catenin signaling [11,13].

## 4. Role of Klotho in Diabetes and Diabetic Nephropathy

The disruption of phosphate and calcium metabolism, oxidative stress, inflammation, the fibrotic process, and an increase in the ratio of β-cell and podocyte loss through apoptosis can result in pathologies that resemble premature aging. Klotho protects cells against accelerated aging and damage during the course of DM and diabetic nephropathy [13,18]. Moreover, a reduction of plasma and urine levels of circulating soluble Klotho was observed during the aging process in cells with short telomeres or stress-induced premature senescence, widespread tissue injury, inflammation, oxidative stress and vascular calcification, which occur during the course of chronic kidney disease (CKD). Additionally, in patients with CKD, low levels of calcitriol (i.e., the bioactive form of vitamin D3) were found to intensify renal Klotho deficiency [11]. Notably, the leading cause of CKD in Western countries is diabetic nephropathy [21]. Low levels of Klotho mRNA and protein were also expected to be observed in diabetic nephropathy. This assumption was confirmed in diabetic mice. In two models of T1DM, Klotho deficiency promoted the apoptosis of insulin-producing β-cells, which were protected against this process after Klotho overexpression. Furthermore, Klotho improved the β-cell function and prevented the development of type 2 DM. Low plasma, but not urine, levels of Klotho predicted the progression of nephropathy in T2DM patients and were negatively correlated with a decrease in the glomerular filtration rate (GFR) [13,22]. Moreover, diabetes-induced proteinuria, oxidative stress (reflected by intracellular ROS levels), podocyte injury, and apoptosis that was caused by protein kinase Cα (PKCα) activation were aggravated by Klotho deficiency and partially ameliorated by Klotho overexpression [23]. Both plasma and urine levels of soluble Klotho were lower in intrinsic AKI patients compared with prerenal AKI patients, together with an increase in levels of proinflammatory ligands (i.e., S100A8/A9 calgranulins) of Toll-like receptor 4 (TLR4) [24]. Therefore, plasma or urine levels of the Klotho protein were proposed to serve as a biomarker of early kidney injury in diabetic patients.

## 5. Immune Response in Diabetes and Diabetic Nephropathy

**Figure 1:**
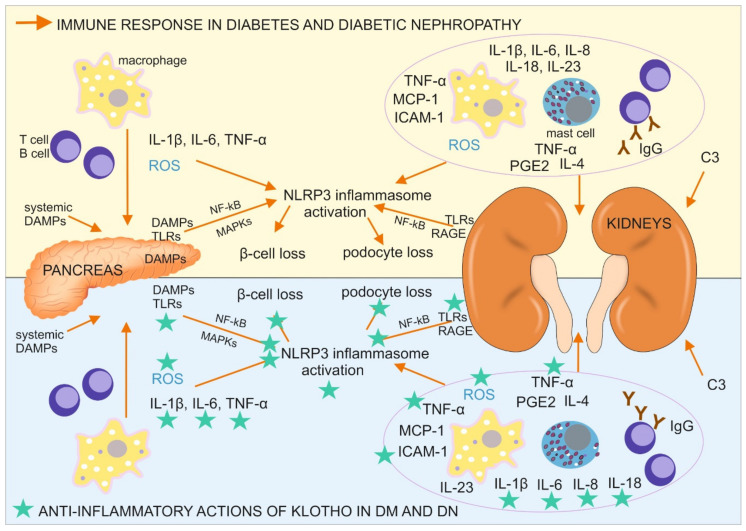
Immune response in diabetes mellitus (DM) and diabetic nephropathy (DN) with a designation of its tissue location, together with antiinflammatory actions of Klotho, directed on the cells, pathways, and proteins involved in the described immune response. C3, complement component 3; DAMPs, danger associated molecular patterns; ICAM-1, intercellular adhesion molecule 1; IgG, immunoglobulin G; IL, interleukin; MAPKs, mitogen-activated protein kinases; MCP-1, monocyte chemoattractant protein 1; NF-κB, nuclear factor κ-light-chain-enhancer of activated B cells; NLRP3 inflammasome, a type of inflammasome that contains NACHT, LRR, and PYD domain-containing protein 3; PGE2, prostaglandin E_2_; RAGE, receptor for advanced glycation endproducts; ROS, reactive oxygen species; TLR, Toll-like receptor; TNF-α, tumor necrosis factor α.

Type 1 DM is triggered by unknown environmental factors and develops in individuals with a polygenetic predisposition through the destruction of insulin-producing pancreatic β-cells. The damage to these cells is caused by impairments in the immune response and local inflammation (Figure 1), including the infiltration of pancreatic islets by M1 macrophages, T and B lymphocytes (CD8^+^ and CD4^+^ T cells and CD20^+^ B cells), and the expression of proinflammatory cytokines, especially interleukin 1β (IL-1β) and TNF-α. High levels of proinflammatory and profibrotic IL-6 are produced in initial stages of T1DM. Moreover, IL-6-inducible autoimmunity-related gene (*HIP*/*PAP*) has been shown to be expressed in the pancreas in patients with T1DM, providing further evidence that IL-6 participates in the autoimmune process in type 1 DM [25].

Type 2 DM develops during the course of a prolonged high-glucose diet and consists of impairments in pancreatic insulin secretion and a lower cellular response to insulin stimulation. These metabolic changes are accompanied by a low-grade inflammatory process in adipose tissue, the liver, and pancreatic islets, in which IL-1β plays a major role [12]. The prolonged upregulation of IL-1β leads to an increase in serum insulin levels, which promotes glucose uptake by macrophages, which infiltrate the pancreatic tissue, with an upregulation of their proinflammatory activity, including the production of ROS and initiation of the generation of the NLRP3 (NOD-, LRR-, and pyrin domain-containing protein 3) inflammasome [26]. The resulting inflammation in the pancreas leads to β-cell loss, whereas the immune reaction in adipocytes and liver cells, especially IL-1β–NLRP3 inflammasome pathway activation, results in a lower insulin response [8]. NLRP3 inflammasome activation in the pancreas in diabetic patients is instigated by oxidative stress and systemic, and islet tissue-derived danger associated molecular patterns (DAMPs), including high-mobility group box 1 (HMGB1), heat shock protein 70 kDa (HSP70), islet amyloid polypeptide, and fatty acids (e.g., palmitate). These DAMPs can bind TLR2 and TLR4, which are expressed by cells in pancreatic islets and pancreatic macrophages, trigger the activation of NF-κB and mitogen-activated protein kinases (MAPKs), and further promote NLRP3 inflammasome activation. Another DAMP, thioredoxin-interacting protein (TXNIP), was shown to initiate posttranslational activation of the NLRP3 inflammasome in T2DM and trigger IL-1β production, resulting in the inflammation of pancreatic islets and apoptosis of β cells [26]. Another interleukin, IL-6, was shown to participate in the development of type 2 DM. IL-6 levels were higher in patients with T2DM, which exacerbated insulin resistance and caused atherosclerosis through its role in accelerating inflammation [25].

This multiorgan inflammatory reaction, accompanied by higher levels of its marker C-reactive protein (CRP), causes pathological changes in the microvascular circulation in the kidneys, macrophage infiltration of this organ, and the local production of a wide range of proinflammatory cytokines and mediators that are detectable beginning in early stages of diabetic nephropathy in serum and peripheral blood cells in affected individuals.

Inflammatory macrophage accumulation in the interstitium of the kidney correlates with glomerulosclerosis, tubular atrophy, interstitial fibrosis, albuminuria, and the loss of function of this organ. Albuminuria leads to an increase in the uptake of proteins in proximal tubules of the nephron, thereby increasing the tubular protein load, which acts as a trigger of the even greater activation of signaling cascades that cause inflammation and fibrosis in the tubule-interstitial region [6]. M1 macrophage accumulation has been observed in the kidneys in diabetic nephropathy patients, in addition to the renal presence and degranulation (with prostaglandins and cytokines, such as IL-4 and TNF-α) of mast cells that correlate with disease progression. Further evidence of the direct participation of T cells and B cells in the development of diabetic nephropathy is needed, but IL-17A (which is produced by Th17 cells, which are a kind of T-helper cell) has been shown to have the ability to protect diabetic kidneys against the inflammatory response. Conversely, immunoglobulins, especially IgG in complex with hyperglycemia-induced AGEs, which are produced by B cells are abundantly present in diabetic animals and associated with the development of albuminuria [8,27]. Furthermore, a common feature of diabetic nephropathy is the presence of a linear smear, stained for IgG, along glomerular (GBM) and tubular basement membranes, which could be a result of an increased antigen and autoantigen load due to frequent infections occurring in DN individuals, high level of AGEs and administration of exogenous insulin [8,27]. However, it might not be simple a cause of IgG-based immune complexes accumulation in a part of glomerular filtration barrier (GBM) due to its high molecular weight, since it is also present in tubular basement membrane. The intensity of the aforementioned anti-IgG staining increases during the course of diabetic nephropathy and is associated with glomerular deposition of the C3 component of the complement system. Many studies have shown that all three pathways of complement activation may be involved in kidney injury in diabetic nephropathy through C1q, C3, and C5 components (i.e., the classic pathway), mannose binding lectin (MBL) activator (i.e., the lectin pathway), and TLR2 and TLR4 (i.e., the alternative pathway), which can increase complement factor B synthesis and release by macrophages. The role of B cells in diabetic nephropathy can be furtherly accelerated by their influence on macrophage activation, in which macrophages possess receptors for Fc fragment of IgG (FcRs), C3 protein, and AGEs (receptor for AGEs-RAGE) [8].

Other receptors also contribute to an accelerated immune response, albuminuria, and podocyte damage in diabetic nephropathy. These are intracellular nucleotide-binding oligomerization domain (NOD)-like receptors (NLRs; e.g., NOD2) that initiate NLRP3 inflammasome formation and the expression of proinflammatory molecules by immune cells (e.g., IL-1β, IL-6, IL-8, IL-18, TNF-α, and monocyte chemoattractant protein 1 (MCP-1), also called chemokine-CC motif ligand (CCL2)) and intracellular adhesion molecule 1 (ICAM-1) [8]. Notably, some of these receptors (e.g., TLR4, RAGE, some FcRs, and NOD2) are not only expressed on/in immune cells but also on/in podocytes (continuously or upon stimulation) where they activate antigen (e.g., AGEs) phagocytosis, antigen presentation with major histocompatibility complex (MHC) molecules to T cells, and inflammasome formation, leading to apoptotic podocyte loss [28,29]. The production of proinflammatory molecules can also occur in mesangial cells, which possess receptors for Fc fragments of immunoglobulin G (FcγRs), which are crucial for antigen recognition and engulfment during phagocytosis. Mice with FcγR deficiency were protected against inflammation, glomerular damage, and albuminuria in diabetic nephropathy [8]. Furthermore, the levels of other inflammation-associated receptors (e.g., TLR2, TLR4, and RAGE) were elevated in diabetic nephropathy kidney tissue, together with DAMPs, such as AGEs, HMGB1, HSP70, and S100 calgranulins (e.g., S100A8 and S100A9), triggering their activation. The expression and function of TLR4 also increased in monocytes in diabetes patients [30]. Signaling through these TLRs is associated with myeloid differentiation factor 88 (MyD88) protein stimulation. Signaling through TLR4 and RAGE leads to NF-κB activation and the subsequent stimulation of MyD88, p38 MAPK, c-Jun N-amino-terminal kinase (JNK), and (PKCβ). The activation of these signaling pathways leads to the production of many proinflammatory cytokines, chemokines, cellular ligands, growth factors, leukocyte adhesion molecules (e.g., IL-1β, IL-6, IL-23, TNF-α, TGF-β1, MCP-1, macrophage inflammatory protein 1β (MIP-1β), granulocyte monocyte-colony stimulating factor (GM-CSF), and prostaglandin E2 [31]), ROS, nitric oxide (NO), inducible nitric oxide synthase (iNOS), and various receptors [8,31,32]. All of these molecules contribute to inflammation-related kidney injury in diabetic nephropathy.

Further immune response facilitation in diabetic nephropathy is caused by the activation of cytokine, chemokine, and angiotensin receptors that influence signaling through Janus kinase-signal transducer and activator of transcription (JAK-STAT) proteins, the mRNA levels of which correlate with the progression of diabetic nephropathy. Urine levels of MCP-1 increase in DM patients with micro- and macroalbuminuria. Moreover, MCP-1 levels correlate with the urine albumin/creatinine ratio and a decrease in renal function. In diabetic patients with macroalbuminuria, MCP-1 levels are a predictor of the progression of nephropathy. The course of the disease may also be monitored by measuring the level of other inflammation-related molecules, such as soluble TNF receptor-2 (sTNFR-2), which is associated with a decrease in the GFR. A ligand of sTNFR-2, TNF-α, and some of the other aforementioned molecules (e.g., IL-1β, IL-6, PGE2, and TGF-β1) are responsible for interstitial kidney tissue destruction through the generation of fibrotic lesions [8]. Additionally, TNF-α expression in mice was shown to be higher in a wide variety of cells by high glucose levels, AGEs, and many RAGE ligands, thereby promoting the development of obesity-induced insulin resistance [32,33]. Moreover, TGF-β1 and TNF-α decrease the expression of nephroprotective Klotho and PGC-1α proteins, which can protect kidney tissue against many harmful factors, such as ROS, and act against many of the aforementioned inflammatory, fibrotic, and apoptotic processes during the course of diabetes and diabetic nephropathy [34,35].

## 6. Antiinflammatory Actions of Klotho in Diabetes and Diabetic Nephropathy

### 6.1. Klotho Expression Is Downregulated during Inflammation

The downregulation of renal Klotho mRNA and protein expression leads to an increase in inflammation in the kidneys in diabetic mice [36]. Additionally, in mice with Klotho haploinsufficiency (Klotho^+/−^), a spontaneous and persistent increase in blood pressure was observed, which was accelerated by a high-salt diet. High salt intake in Klotho^+/−^ mice significantly increased the expression of MCP-1 in the kidneys and caused the infiltration of this organ with macrophages and T lymphocytes [37]. Prolonged systemic hypertension highly contributes to endothelial injury in the kidneys. High blood pressure is a common complication of diabetes that occurs through volume expansion in blood vessels in both type 1 and type 2 DM [6]. A decrease in the expression of Klotho, observed in DM and DN [13,22], was also found in CD4^+^ lymphocytes (T-helper cells) in healthy elderly individuals and individuals with rheumatoid arthritis, a chronic autoimmune disorder that causes joint inflammation. In T-helper cells in these patients, Klotho was downregulated at the mRNA, protein, and enzyme activity (β-glucuronidase) levels. The role of Klotho in these cells would be to contribute to an antiinflammatory process, present in young and healthy subjects but lowered together with senescence and in inflammatory disorders, such as rheumatoid arthritis, diabetes, and diabetic nephropathy. This hypothesis has been supported by the fact that a reduction of Klotho expression and activity in T-helper cells in elderly individuals and rheumatoid arthritis patients occurred concomitantly with the downregulation of costimulatory CD28 molecules in these cells, which depend on higher levels of proinflammatory TNF-α [38]. As mentioned above, TNF-α was shown to decrease the expression of Klotho protein and lead to fibrosis in renal tissue [2]. On the contrary, Klotho suppresses TNF-α expression and the TNF-α-induced production of many inflammation-related molecules [39,40].

### 6.2. Klotho Induces Antiinflammatory Reactions

Klotho also stimulates the production of antiinflammatory factors. In human monocytes that were stimulated with lipopolysaccharide (LPS) to induce an immunosenescent-like phenotype, Klotho production, induced by calcitriol and transfection of cells with Klotho gene-possessing plasmid, upregulated the secretion of IL-10 [40]. IL-10 is also referred to as a cytokine synthesis inhibitory factor that inhibits the expression of many proinflammatory cytokines, such as TNF-α. The upregulation of IL-10 results from Klotho-mediated activation of the JAK2/STAT3 signaling axis. IL-10-induced TNF-α inhibition in these cells could be attributable to the destabilization of TNF-α mRNA expression via the suppression of p38 MAPK activation and the inhibition of NF-κB and RNA-binding Hu-antigen receptor (HuR) expression, all of which induce an inflammatory reaction and tissue injury during the course of diabetes and diabetic nephropathy. DNA damage in the aforementioned LPS-treated monocytes was less severe when Klotho expression was induced in them. The cells promptly resumed a normal proliferation rate and regained immune functionality, with adhesion and phagocytic activity [40,41].

### 6.3. Klotho Suppresses Proinflammatory NF-κB Activation

Klotho and TNF-α have opposite effects on NF-κB activation through inhibition vs. promotion of the phosphorylation of RelA at Ser^536^, which is a NF-κB p65 subunit. The phosphorylation of RelA at Ser^536^, which was induced by TNF-α, caused NF-κB activation and the expression of several proinflammatory cytokines that are relevant to diabetes development, including TNF-α, which were found to be higher in the renal cortex in db/db diabetic mice and correlated with the duration of diabetes [32]. TNF-α and TNF-like weak inducer of apoptosis (TWEAK), through NF-κB activation, caused RelA to bind the Klotho promoter and resulted in the deacetylation and inhibition of Klotho expression [42]. Conversely, both transmembrane and soluble Klotho suppress TNF-α-induced NF-κB activation by inhibiting RelA phosphorylation at Ser^536^ and its further recruitment on IL-6, IL-8, MCP-1, and RANTES (chemokine (C-C motif) ligand 5 (CCL5)) promoters, thereby inhibiting the production of these proinflammatory molecules and protecting kidney tissue against inflammation-associated damage (Figure 1) [32]. Klotho was also suggested to inhibit NF-κB activation through its influence on the main inhibitor of RelA, IκBα [43]. As mentioned above, Klotho may also restrain the action of NF-κB by inhibiting its translocation to the nucleus, decreasing its DNA-binding activity, and blocking initiation of the TNF-α-induced phosphatidylinositol 3-kinase (PI3K)/protein kinase B (Akt) pathway, which is involved in cellular inflammatory responses to stimuli [13,32,44]. Furthermore, as demonstrated in vitro, by transferring exosomes from cells with Klotho overexpression to pancreatic cell cultures, in which acute pancreatitis has been induced, Klotho inhibited NF-κB activation and inflammation in these cells and inhibited apoptotic pancreatic cell loss [44]. In cultured macrophages, Klotho suppressed the lipopolysaccharide (LPS)-induced activation of NF-κB by upregulating HSP70 levels, which are elevated in diabetic nephropathy kidney tissue. HSP70 is located perinuclearly and has been proposed to prevent the translocation of NF-κB to the nucleus [39].

### 6.4. Klotho Inhibits TLR4 Signaling and Related Oxidative Stress

Klotho has also been shown to cause the degradation of TLR4, the receptor for LPS, via a deglycosylation-associated proteolytic process and thus attenuate LPS-induced acute kidney injury [45]. Lipopolysaccharide-induced proinflammatory signaling acts through NF-κB activation and causes the elevation of TLR4 levels in macrophages, renal epithelial cells, and inflamed kidneys, together with the repression of Klotho expression. Therefore, Klotho and TLR4 inhibit one another [45]. Notably, TLR4 is a receptor for exogenous ligands that are associated with pathogens (i.e., pathogen-associated molecular patterns (PAMPs); e.g., LPS) and also endogenous ligands (DAMPs; e.g., free fatty acids) that are elevated in obese individuals. Obesity and high glucose intake are firmly associated with diabetes. TLR4 mRNA expression was higher in patients with T2DM than in healthy individuals and shown to be involved in the development of tubular inflammation in diabetic nephropathy [30,46]. Moreover, the expression of downstream factors of TLR4, such as MyD88 and NF-κB, was significantly elevated in cells that were exposed to high glucose concentrations [30]. Through binding of the aforementioned ligands (e.g., PAMPs and DAMPs), TLR4 contributes to the development of insulin resistance and inflammation. TLR4 expression in insulin target tissues causes activation of the inflammation-associated kinases JNK, IκB kinase (IKK), and p38 MAPK, which directly inhibit insulin signal transduction through the phosphorylation of insulin receptor substrate (IRS) on serine residues. TLR4 activation also increases the transcription of proinflammatory genes and the production of many cytokines (e.g., IL-1β and IL-18), chemokines, and ROS that promote further insulin desensitization via paracrine and systemic effects. Therefore, the inhibition of TLR4 by Klotho contributes to the protection of tissues against inflammation-associated injury and insulin-resistance, especially because TLR4 is involved in generating oxidative stress [30,47]. This has a great impact on disease progression in diabetes and diabetic nephropathy because of an increase in the expression of TLR4 in these pathological states and because hyperglycemia causes significant oxidative stress by increasing PKC-triggered NADPH oxidase activity and ROS levels and impairing NO metabolism [8,30,48]. The administration of antioxidants (e.g., thioredoxin) reduced the expression of TLR4 and its downstream markers of inflammation similarly to the overexpression of Klotho [30].

### 6.5. Klotho Reduces Oxidative Stress by Inhibiting IGF-1 Signaling and NLRP3 Inflammasome Activation

Klotho lowers oxidative stress through its impact on TLR4 and through the aforementioned inhibition of the IGF-1 signaling cascade and subsequent activation of FoxO transcription factors, the induction of MnSOD expression, and ROS removal [48]. It also reduces oxidative stress by suppressing TXNIP-dependent activation of the NLRP3 inflammasome in macrophages through an enhancement of FGF23 signaling. TXNIP is also known as a vitamin D3-upregulated protein because the bioactive form of vitamin D3 (calcitriol) increases its expression. FGF23/Klotho pathway activation suppresses the expression of 1-α-hydroxylase, an enzyme that converts 25-hydroxycholecalciferol to 1,25-dihydroxycholecalciferol (calcitriol), and increases the expression of 24-hydroxylase, thereby promoting the degradation of calcitriol. Additionally, the pretreatment of macrophages with Klotho and FGF23 blocked the calcitriol-induced enhancement of IL-1β release. During the inflammatory process, Klotho and FGF23 do not always act in concert. Excess FGF23 that occurs during inflammation inhibits Klotho expression in the kidneys. Additionally, FGF23, acting alone, promotes macrophage polarization toward their proinflammatory M1 type and inhibits the macrophage transition from the M1 type to an antiinflammatory M2 type, thereby increasing morbidity and mortality that are associated with CKD. Klotho was found to regulate the actions of FGF23 on the immune response [36]. Therefore, Klotho, alone or together with FGF23, inhibits the effects of vitamin D3 and TXNIP on IL-1β production and NLRP3-inflammasome activation, with further ROS accumulation [49]. By inhibiting NLRP3-inflammasome activation, Klotho prevents maturation of the proinflammatory cytokines IL-1β and IL-18, cell death, and the loss of pancreatic islet mass and lowers insulin resistance [26].

### 6.6. Klotho Reduces Leukocyte Infiltration of the Kidneys, Renal Injury, and Fibrosis

By inhibiting TXNIP, Klotho downregulates the production of ICAM-1, a TXNIP-, IL-1β-, and TNF-α-stimulated proinflammatory factor that promotes leukocyte infiltration into tissues [49]. ICAM-1 deficiency reduced leukocyte accumulation, tubular and glomerular injury, interstitial fibrosis, and albuminuria in mice with DM [8]. An increase in mRNA levels of ICAM-1 and retinoic acid-inducible gene I (RIG-I) was observed in Klotho-haploinsufficient mice, with resulting renal damage [50]. Furthermore, in aged cells and mice, Klotho inhibited endothelial activation that was associated with senescence and inflammation through an interaction with RIG-I. In vitro and in vivo studies have shown that Klotho inhibits RIG-I, which promotes NF-κB activation and the expression of two important mediators of inflammation, IL-6 and IL-8, and is a receptor that is involved in the regulation of LPS-mediated endothelial activation [50]. IL-6, IL-8, and their receptors play a crucial role in the development and persistence of the senescence-like phenotype through their contribution to the maintenance of inflammatory states. Additionally, in Klotho-deficient mice, the expression, secretion, and accumulation of IL-6 are higher in serum compared with wildtype animals [51].

Podocytes are key cells that maintain proper renal filtration and are the only cells in renal tissue that express IL-6 receptors, and this expression is elevated by proinflammatory stimulation. Podocytes are also a well-known source of IL-6, but only after being exposed to proinflammatory mediators, such as LPS, TNF-α, and IL-1β, and high glucose levels. Therefore, IL-6 appears to be an important factor in the development of a local inflammatory response in the kidneys in diabetic nephropathy, which is counteracted by calcitriol and Klotho protein [25]. 

IL-6 was also found to trigger proximal tubular epithelial cells to generate collagen I fibers and subsequently accelerate tubulointerstitial fibrosis [25]. Together with IL-6, IL-8 (after being induced by IL-1) has been shown in humans to be involved in renal interstitial fibrosis [52]. The fibrotic process in the kidneys in patients with diabetic nephropathy may also be induced by TGF-β1 and the Wnt/β-catenin pathway, both of which are inhibited by Klotho. Soluble Klotho inhibits TGF-β1 signaling. It binds to type II TGFβ receptors, thus preventing TGF-β1 attachment to its cell-surface receptors. The downregulation of Klotho expression aggravates renal interstitial fibrosis through the upregulation of TGF-β1 and activation of canonical Wnt (Wnt/β-catenin) signaling, which was observed in CKD patients [39,48]. Klotho can bind several Wnt proteins (e.g., Wnt1, Wnt4, and Wnt7a), inhibit their activity, and prevent renal inflammation and fibrosis by protecting various cells from premature senescence [48].

Therefore, Klotho may have therapeutic potential as an antiaging molecule and an alleviator of the effects of DM and DN by activating antiinflammatory processes, and inhibiting proinflammatory and profibrotic processes (Table 1). 

## 7. Klotho May Serve as a Key Molecule in Immunotherapy of Diabetes and Diabetic Nephropathy

Several immunotherapeutic treatment approaches for type 1 and type 2 DM, diabetic nephropathy, and other kidney disorders have been proposed. One such approach, in which diabetic rodents were treated with immunosuppressants (e.g., mycophenolate mofetil and sirolimus), demonstrated the usefulness of immunotherapy for DM and diabetic nephropathy by lowering the degree of renal inflammation. These drugs reduced development of the inflammatory reaction in the kidneys and protected against podocyte loss, glomerulosclerosis, and albuminuria. Another strategy to diminish renal inflammation and injury in an animal model of diabetic nephropathy was to inhibit NLRP3-inflammasome activation. A superoxide dismutase mimetic (MitoTempo) was used in this study to reduce oxidative stress and shown to downregulate NLRP3-inflammasome activation and renal injury. NLRP3-inflammasome activation upregulates expression of the proinflammatory cytokines IL-1β and TNF-α [8]. Based on knowledge of their destructive effects on tissues during accelerated inflammation in DM, the use of some of their antagonists was tested to improve insulin secretion in patients with T1DM. However, despite promising initial results, these drugs were not successful in Phase 2 clinical trials. For T2DM, clinical trials reported that IL-1β antagonists (IL-1Ra and anti-IL-1β antibodies) had promising results with regard to attenuating inflammation. In patients with CKD, treatment with IL-1 soluble receptor trap (rilonacept) decreased CRP levels and reduced vascular oxidative stress [34]. Notably, Klotho protein exerts inhibitory actions on NLRP3-inflammasome activation, IL-1β and TNF-α expression, and oxidative stress [49].

Targeting other signaling pathways that are inhibited by Klotho has shown promising results with regard to improving health in patients with DM and diabetic nephropathy. Strategies of interfering with MyD88 signaling complex formation were tested in a mouse model of type 1 DM. Small molecules, such as L6H21, were shown to suppress the development of T1DM and kidney injury in these rodents by affecting MyD88 signaling and the expression of many proinflammatory cytokines (e.g., IL-1β, IL-6, and TNF-α) in kidney tubular cells. Furthermore, the abrogation of p38 MAPK signaling activation by GS-4997 in the kidneys in diabetic mice halted the progression of diabetic nephropathy and reduced the macrophage infiltration of these organs and the expression of genes that encode MCP-1 and TNF-α. Therefore, GS-4997 has been proposed as a therapeutic agent for the treatment of diabetic nephropathy in patients with T2DM. Similarly, the lower expression of MCP-1 and ICAM-1, which are inhibited by Klotho, reduced the accumulation of macrophages in the kidneys in animals with diabetic nephropathy and decreased tubulointerstitial injury after PKC-β inhibition by ruboxistaurin [8]. Additionally, in a rat model of streptozotocin-induced diabetes-associated renal fibrosis, a combination of metformin and a plant extract (from *Abelmoschus manihot* (L.) *medic*), traditionally used against CKD, was proved to act in part via the Klotho/TGF-β1/p38MAPK signaling pathway and to significantly lower the weight of the rats with DN, reduce their blood glucose and urine protein level, and the degrees of renal tubule damage, apoptosis, glomerulopathy, and renal fibrosis [57]. A preservation of the Klotho level with simultaneous decrease in proteinuria in DN was also observed after administration of pentoxifylline in a phase 2 of the clinical trial [58].

Furthermore, a few studies have evaluated the therapeutic effects of recombinant Klotho and the stimulation of Klotho overexpression (e.g., by calcitriol, peroxisome proliferator-activated receptor γ [41], substances like pioglitazone [7] and Klotho gene delivery through a viral vector), which downregulated oxidative stress, ameliorated the high glucose-induced injury of renal glomerular endothelial cells in a model of diabetic nephropathy, suppressed diabetes-induced renal hypertrophy, and exerted other immunotherapeutic effects in diabetic nephropathy [12,16,44,59]. When podocytes that were cultured in a medium with high glucose concentrations were incubated with high Klotho protein levels, podocyte injury was significantly attenuated compared with podocytes that were not treated with Klotho. In these cells, Klotho lowered the expression of proapoptotic markers, preventing podocyte loss. Klotho also attenuated the increase in levels of profibrotic markers in high glucose-exposed podocytes [41]. These results were confirmed by another study, in which the administration of soluble Klotho protein attenuated renal fibrosis, inhibited the expression of profibrotic markers and TGF-β1 target genes, and suppressed the Wnt signaling pathway [60]. Additionally, healthy mouse urine-derived extracellular vesicles containing Klotho accelerated renal recovery, stimulating tubular cell proliferation, reducing the expression of inflammatory and injury markers, and restoring endogenous Klotho loss in a murine model of acute injury generated by glycerol injection [61].

Therefore, preclinical data on Klotho have shown its potential usefulness as an early biomarker for DN development and its progression, and a possible, safe and effective agent in ameliorating diabetic kidney function [48,60,62,63]. However, technological issues in measuring soluble Klotho in blood and urine need to be addressed for its standardization and repeatability of measurements between laboratories for Klotho to be considered as a biomarker of the DN course. Additionally, multiple gaps in the knowledge about the mechanisms of Klotho action exist, especially in human organisms. Up to now, no clinical studies on recombinant Klotho administration in humans were reported [62,63]. Additionally, no analyses on the effective dose of Klotho or the needed frequency of its administration were performed, which must be done before introducing Klotho as a promising agent in immunotherapy for diabetes and diabetic nephropathy.

## Figures and Tables

**Table 1:** Actions of Klotho on various cells, pathways, and proteins that are involved in inflammatory and fibrotic processes in diabetes and diabetic nephropathy, with their reciprocal actions on the Klotho protein.

Cell/Pathway/Protein	Action of Klotho on the Cell/Pathway/Protein	Action of the Cell/Pathway/Protein on Klotho	Connections with Other Cells/Pathways/Proteins	Reference
T cells	Klotho downregulation causes T-cell infiltration of kidneys			[37]
T-helper cells (CD4^+^ lymphocytes, CD28 molecule)	Klotho downregulation in CD4^+^ lymphocytes is associated with lower levels of CD28 molecule through an increase in TNF-α		TNF-α	[38]
Macrophages	Klotho downregulation causes macrophage infiltration of kidneys			[37]
MCP-1 (CCL2)/CCR2, ICAM-1	Klotho downregulation increases the expression of MCP-1 and ICAM-1 in the kidneys Klotho inhibits TXNIP-mediated expression of ICAM-1		PKC, TXNIP, IL-1β, TNF-α, RIG-I	[8,37,49,50]
Complement system		Proteins of the complement system (C1, C5a), released during inflammation, inhibit Klotho expression		[53]
PI3K/Akt	Klotho blocks TNF-α-induced PI3K/Akt pathway to restrain NF-κB activation		TNF-α, NF-κB	[13]
TNF-α, TWEAK	Klotho inhibits TNF- α actions on the intensification of inflammatory processes	TNF-α and TWEAK inhibit Klotho through NF-κB activation	PGC-1α, NF-κB	[39,42]
NF-κB and its p65 subunit (RelA)	Klotho inhibits NF-κB activity	NF-κB inhibits Klotho expression	p38 MAPK, JNK, PKC, TNF-α, IL-6, IL-8, IL-10, MCP-1, RANTES (CCL5)	[8,32,39,42,54]
IκBα	Klotho modulates IκBα function to inhibit RelA activation		NF-κB, RelA	[43]
HSP70	Klotho increases HSP70 levels, which inhibit NF-κB activation		NF-κB	[39]
TLR4	Klotho induces proteolytic degradation of TLR4	TLR4 induces downregulation of Klotho expression	RIG-I, IL-1 β, MyD88, NF-κB, JNK, IKK, p38 MAPK, IRS	[8,45]
RIG-I	Klotho inhibits RIG-I		NF-κB, IL-6, IL-8, TLR4	[50,51]
IL-6, IL-8	Klotho inhibits IL-6 and IL-8 through RIG-I and NF-κB suppression		RIG-I, NF-κB	[50,51]
IL-1β, NLRP3	Klotho inhibits vitamin D3 and TXNIP effect on IL-1β production and NLRP3-inflammasome activation		calcitriol, TXNIP	[49]
IL-10	Klotho increases IL-10 secretion		JAK2/STAT3, p38 MAPK, HuR, TNF-α, NF-κB	[40]
Vitamin D3 in its bioactive form (calcitriol)	FGF23/Klotho pathway activation suppresses calcitriol production and promotes its degradation	Calcitriol stimulates expression of Klotho and FGF23	FGF23	[40,49,55]
TGF-β1	Klotho binds to type II TGFβ receptors, thus inhibiting TGF-β1 signaling		Wnt, PGC-1α, MAPK, NF-κB, Smads	[54,56]
Wnt/β-catenin	Klotho binds several Wnt protein family members, thus inhibiting canonical Wnt signaling		TGF-β1, PGE2	[48]
ROS	Klotho inhibits IGF-1 signaling cascade, thus causing the activation of FoxO transcription factors, induction of the expression of MnSOD, and removal of ROS Klotho suppresses TXNIP-dependent activation of the NLRP3 inflammasome in macrophages through the enhancement of FGF23 signaling		TLR4, PGC-1α, IGF-1, FoxO, MnSOD, TXNIP, NLRP3, FGF23	[48]
NO	Klotho modulates NO metabolism and prevents its impairment			[48]

CCR2, C-C chemokine receptor type 2; FGF23, fibroblast growth factor 23; FoxO, forkhead box transcription factors; HSP70, heat shock protein 70 kDa; HuR, Hu-antigen receptor; ICAM-1, intercellular adhesion molecule 1; IGF-1, insulin-like growth factor 1; IKK, IκB kinase; IL, interleukin; IRS, insulin receptor substrate; IκBα, nuclear factor of κ light polypeptide gene enhancer in B-cells inhibitor α; JAK2/STAT3, proteins of the Janus kinase-signal transducer and activator of transcription signaling pathway; JNK, c-Jun N-amino-terminal kinase; MBL, mannose binding lectin; MCP-1, monocyte chemoattractant protein 1, also referred to as chemokine (C-C motif) ligand 2 (CCL2); MnSOD, superoxide dismutase; MyD88, myeloid differentiation factor 88; NF-κB, nuclear factor κ-light-chain-enhancer of activated B cells; NLRP3 inflammasome, a type of inflammasome that contains NACHT, LRR, and PYD domain-containing protein 3; NO, nitric oxide; p38 MAPK, p38 mitogen-activated protein kinase; PGC-1α, peroxisome proliferator-activated receptor γ coactivator 1-α; PGE2, prostaglandin E_2_; PI3K/Akt, phosphatidylinositol 3-kinase/protein kinase B; PKC, protein kinase C; RANTES, regulated on activation, normal T cell expressed and secreted, also referred to as chemokine (C-C motif) ligand 5 (CCL5); RelA, nuclear factor NF-κB p65 subunit; RIG-I, retinoic acid-inducible gene I; ROS, reactive oxygen species; TGF-β1, transforming growth factor β1; TLR, Toll-like receptor; TNF-α, tumor necrosis factor α; TWEAK, tumor necrosis factor-like weak inducer of apoptosis; TXNIP, thioredoxin-interacting protein.

## Data Availability

Data sharing not applicable.
